# Point-of-sale Naloxone: Novel Community-based Research to Identify Naloxone Availability

**DOI:** 10.5811/westjem.7.2020.47252

**Published:** 2020-08-24

**Authors:** Travis Olives, Laurie A. Willhite, Samantha C. Lee, Danika K. Evans, Ashley Jensen, Hsiao-Ting Regelman, Eric S. McGillis

**Affiliations:** * Hennepin Healthcare, Department of Emergency Medicine, Minneapolis, Minnesota; † Hennepin Healthcare, Minneapolis, Minnesota; ‡ University of Calgary, Department of Emergency Medicine, Calgary, Alberta, Canada; § Minnesota Poison Control System, Minneapolis, Minnesota

## Abstract

**Introduction:**

Expanding naloxone availability is important to reduce opioid-related deaths. Recent data suggest low, variable urban naloxone availability. No reports describe naloxone availability at the point of sale (POSN). We characterize POSN without prescription across a Midwestern metropolitan area, via a unique poison center-based study.

**Methods:**

Pharmacies were randomly sampled within a seven-county metropolitan area, geospatially mapped, and distributed among seven investigators, who visited pharmacies and asked, “May I purchase naloxone here without a prescription from my doctor?” Following “No,” investigators asked, “Are you aware of the state statute that allows you to dispense naloxone to the public under a standing order?” Materials describing statutory support for POSN were provided. Responses were uploaded to REDCap in real time. We excluded specialty (veterinary, mail order, or infusion) pharmacies *a priori*. POSN availability is presented as descriptive statistics; characteristics of individual sites associated with POSN availability are reported.

**Results:**

In total, 150 pharmacies were prospectively randomized, with 52 subsequently excluded or unavailable for survey. Thus, 98 were included in the final analysis. POSN was available at 71 (72.5%) of 98 pharmacies. POSN availability was more likely at chain than independent pharmacies (84.7% vs 38.5%, p<0.001); rural areas were more commonly served by independent than chain pharmacies (47.4% vs 21.5%, p = 0.022). Five chain and five independent pharmacies (18.5% each) were unaware of state statutory support for collaborative POSN agreements. Statutory awareness was similar between independent and chain pharmacies (68.8% vs 54.6%, p = 0.453). Rationale for no POSN varied.

**Conclusion:**

POSN is widely available in this metropolitan area. Variability exists between chain and independent pharmacies, and among pharmacies of the same chain; awareness of statutory guidance does not. Poison centers can act to define local POSN availability via direct inquiry in their communities.

## INTRODUCTION

Opioids continue to account for a large proportion of drug-related deaths in the United States, and were involved in over half of all deaths related to drug overdose from 2013–2017.^1^ Nearly 10% of all substances reported in fatal drug exposures reported to US poison centers were attributed to opioids in 2017, making them the second most commonly cited exposure category involved in overdose deaths.^2^ The importance of efforts to decrease or eliminate morbidity and mortality attributed to opioid use, misuse, and overdose remains a public health priority, within which primary and secondary prevention efforts have been increasingly accompanied by efforts to broaden the distribution of the opioid reversal agent, naloxone.^2^,^3^

Naloxone has long been recognized as a competitive opioid receptor antagonist when administered parenterally or intranasally,^4^–^6^ and remains, in conjunction with the restoration of respiration, the reversal agent of choice for the treatment of acute opioid toxicity. Naloxone is increasingly considered an important component of tertiary prevention and harm reduction in the fight against opioids,^7^ in addition to its utility in the care of individual patients at risk for opioid overdose. Naloxone distribution has been shown to be a cost-effective way to decrease overdose mortality,^8^ and evidence modeling naloxone distribution at a time of increasing fentanyl adulteration suggests a survival benefit to naloxone distribution.^9^ Although penetrance of naloxone prescribing varies,^10^ the practical availability of point-of-sale naloxone (POSN) without a medical prescription remains ill-defined. In a manner analogous to previously controversial but now widely accepted needle-exchange programs to prevent the spread of HIV and other communicable diseases,^11^,^12^ expanded availability of POSN without a medical prescription offers the hope of increased access to a life-saving antidote with fewer acquisition barriers.

Although previous studies have characterized the prevalence of POSN availability without a medical prescription within pre-specified geographic areas,^13^,^14^ to our knowledge none have characterized naloxone availability through organized, in-person assessments at the level of individual pharmacies. The purpose of this study was to define the availability of POSN within a Midwestern metropolitan area, and to describe site characteristics associated with POSN availability.

## METHODS

### Study Design and Setting

This was a cross-sectional study of POSN availability conducted at community pharmacies within a large, seven-county metropolitan area in Minnesota with a total population of approximately 3,000,000. The study was identified as exempt from review by the governing institutional review board. Although the greater metropolitan area is entirely within the seven counties, some rural areas within these counties are also represented. Pharmacy locations were defined as rural if located in a community of fewer than 50,000 people, per the US Census Bureau definition,^15^,^16^ and entirely outside of the US interstate 494/694 ring clearly defining the central metropolitan area. The remainder were defined as urban. This definition served to avoid the inclusion of communities of fewer than 50,000 people, but geographically located immediately contiguous with urban areas.

**Population Health Research Capsule**

What do we already know about this issue?

Changes to legislation have facilitated the availability of point-of-sale naloxone (POSN) in many states.

What was the research question?

What is the prevalence of POSN availability at pharmacies in a large Midwestern metropolitan area?

What was the major finding of the study?

Of 98 pharmacies approached on foot by seven Poison Center professionals, 72.5% offered POSN.

How does this improve population health?

When pharmacies are approached directly, POSN availability varies. This variability persists across chain and independent pharmacies despite statutory awareness

### Study Protocol

The Minnesota State Board of Pharmacy lists 569 operational pharmacies within the metro area. From this list we randomly sampled 150 pharmacies to approach for this study by using a random number generator in Excel 2013 (Microsoft Corporation, Redmond WA) and sorting on the randomly generated numbers to select the first 150 sites. We intended to analyze approximately 100 sites, anticipating limitations to time and resources preventing additional sampling. *A priori* exclusion criteria included known sub-specialization, including mail order, veterinary, and infusion center pharmacies. We then geospatially mapped the remaining pharmacies using arcGIS online 2019 (Environmental Systems Research Institute, Redlands, CA) and divided them by geographic location. Seven investigators, all based at a single, accredited poison center were trained equivalently on approaching pharmacies to inquire about the availability of POSN. These investigators were assigned to evaluate sampled pharmacies within a specified geographic region. To minimize the impact of evolving pharmacy protocols over time, all pharmacies were visited within a 24-hour period, the majority of which occurred over a single morning. Once assigned, investigators approached each pharmacy in person and asked a series of scripted questions:

1. “May I purchase naloxone here without a prescription from my doctor?”
  “No” responses to question 1 were followed by a request to ask the question of the onsite pharmacist for verification purposes, if initially answered by a non-pharmacist. Following an answer of “no,” the response to a follow-up question was recorded:
2. ”Are you aware of the state statute that allows you to dispense naloxone to the public under a standing order?”

To simulate anticipated clinical circumstances, the protocol did not specify that a pharmacist had to be approached and queried. Rather, investigators were instructed to question the employee greeting them at the pharmacy.

Pharmacies were provided with information from the state Board of Pharmacy describing statutory support for collaborative agreements for standing orders for naloxone. Data including pharmacy name, survey responses, and geographic address including county and retail status (chain or independent) were entered into the REDCap mobile app and uploaded in real time to a central REDCap v8.11.3 (Vanderbilt University, Nashville, TN) database. REDCap is a web-based clinical research tool for creating and storing databases.

Chain community pharmacies were substantially over-represented in the initial sample. Because of the imbalance that under-representation of independent pharmacies introduced to the dataset, one third of sites from each chain pharmacy with greater than 10 sites sampled were replaced with randomly selected independent pharmacies using the method described above. We chose one third to maintain prominent representation of community chain pharmacies, which are common throughout the study area, while still affording opportunity for a meaningful comparison with independent pharmacies. Stata/IC 15.1 (StataCorp, College Station, TX) was used to assess the association between pharmacies and POSN availability. We employed Pearson’s χ2 to assess the association between the outcome of interest, POSN availability, and independent variables. Where fewer than five observations per cell were encountered, we employed Fisher’s exact test.

## RESULTS

**Figure 1:**
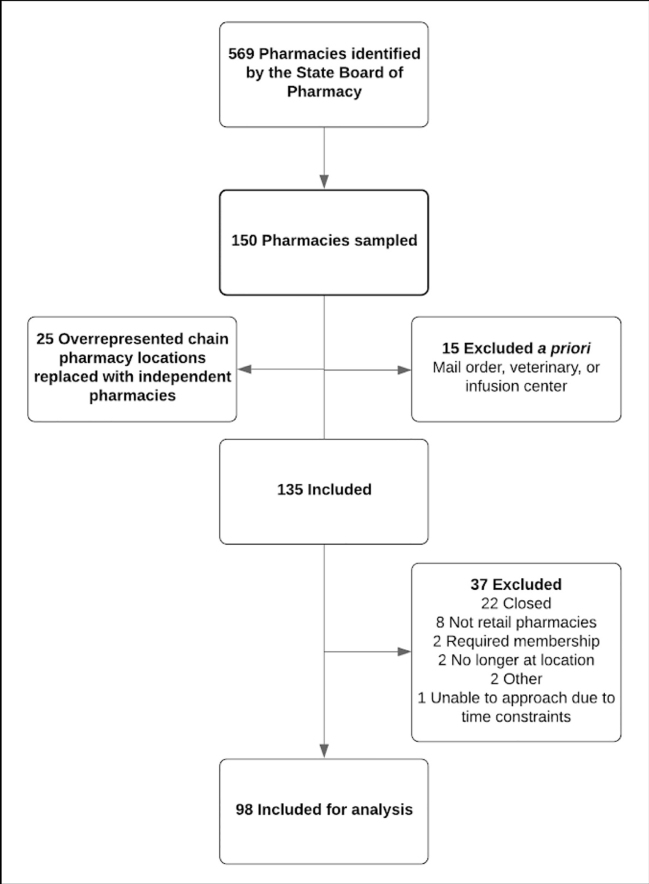
Study flowchart of pharmacies chosen for in-person query regarding over-the-counter naloxone availability.

After *a priori* exclusions of 15 pharmacies for clear evidence of a business model (mail order, veterinary medicine, or infusion center) intended for non-retail or non-human customers, 135 pharmacies (Figure 1) were approached by seven investigators comprised of seven Poison Center staff (five female and two male, of whom four were pharmacists/specialists in poison information, one an emergency medicine resident, one a medical toxicology fellow, and one a medical toxicologist). Median pharmacies approached by a single investigator was 20 (range 16 – 24). Of the 135 pharmacies approached, 37 (27.4%) were excluded due to closure (22, 16.3%); other than community pharmacy (eight, 5.9%); membership requirements (two, 1.5%); not at the described location (two, 1.5%); or other (three, 2.2%). Thus, we included a total of 98 (73.1%) in the study. A single investigator approached 12 (9%) pharmacies the evening prior to the four-hour study period due to time constraints. Of included pharmacies, 75 (76.5%) were urban; the remaining 23 (23.5%) were rural (Table 1). Pharmacies were widely distributed across the seven-county metropolitan area.

**Figure 2:**
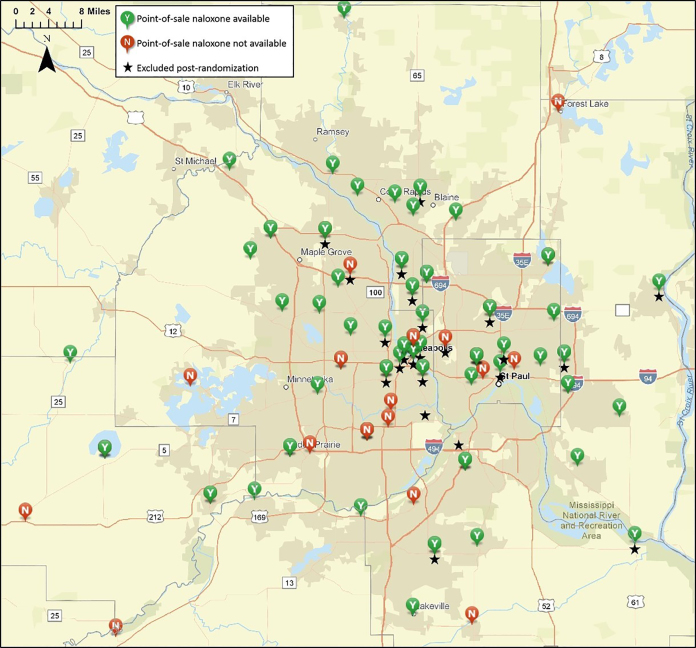
Geospatial distribution of point-of-sale naloxone availability in the Minneapolis/St. Paul, Minnesota metropolitan area.

Naloxone was available at the point of sale at 71 of 98 pharmacies surveyed (72.5%, Figure 2). Pharmacy location was not associated with POSN availability (rural 65.2% vs urban 74.7%, p = 0.375, Table 2). Chain pharmacies were more likely to report POSN availability than independent pharmacies (chain 84.7% vs independent 38.5%, p<0.001), and rural areas were more commonly served by independent pharmacies than chain pharmacies (47.4% vs 21.5%, p = 0.022). Independent pharmacies in rural settings were less likely to offer POSN than chain pharmacies (30.0% vs 92.3%, p = 0.006). Among chain pharmacies with more than one location sampled, POSN availability varied from 66.7–100% (Table 3) across geographic locations.

Among those without POSN availability, 17 (63%) were aware of the state statute allowing for the provision of POSN via collaborative agreement with a prescribing practitioner. No differences in statutory familiarity were apparent when stratified by pharmacy location or retail type (Table 4). Reasons given for not providing POSN included a lack of consumer demand, incomplete stocking plans, no physician collaborator, and refusal to provide naloxone despite availability (“Yes, I am aware of the statute, but I can use my discretion and I won’t be giving it out this time”). Still others indicated that POSN could be provided “if the patient had risk factors for overdose,” or “if the patient was actively overdosing.” A single pharmacy denied POSN availability, was prompted to revisit internal pharmacy guidelines, and then identified POSN as available.

In a post hoc sensitivity analysis to determine the effect of oversampling independent pharmacies, we excluded 25 randomly selected independent pharmacies to account for oversampling. Compared to the overall POSN availability in our primary analysis, POSN availability in our sensitivity analysis suggested that our oversampling of independent pharmacies modestly underestimated availability in this sample (77.8% vs 72.5%). Differences in availability across chain and independent pharmacies remained.

## DISCUSSION

Pharmacy-based POSN is an evolving harm reduction approach to limit morbidity and mortality from opioid overdose. Collaborative naloxone prescribing has developed in parallel with other models of increasing naloxone availability, including point-of-contact,^17^ emergency department-based,^18^ and pharmacist-driven distribution.^19^ The present study suggests that POSN availability is more widespread in this metropolitan area than it was in 10 New Jersey cities that were assessed by telephone survey.^10^ This finding may represent a meaningful difference in the availability of POSN across the two regions, but it also may be attributable to the time lapse of 24 months between the two studies.

Support for POSN via collaborative agreements with medical providers is stipulated in Minnesota statutes (Minn. Stat. §151.37, subd. 13 [2019]), but is predicated on the availability of a collaborating healthcare professional willing to provide a standing order to dispense naloxone. Currently, all states but Wyoming and Kansas have active naloxone-access laws.^20^ Despite this, discrepancies continue to be reported at the point of sale.^21^ Our data suggest that chain community pharmacies were more likely than independent pharmacies to provide POSN, and episodes of within-chain variability were common, with availability ranging from 66.7–100%. This finding is consistent with previously reported variability in POSN availability between chain and independent pharmacies,^14^,^22^ as well as limited practical availability of other medications whose delivery has previously been limited, such as emergency contraception.^23^ Areas served predominantly by independent pharmacies are less likely to have access to POSN than those served by chain pharmacies despite similar awareness of Minnesota statutory support. Previous research has revealed that rural areas of Minnesota are predominantly served by independent pharmacies,^24^ potentially placing rural populations at risk of poorer access to POSN than their urban counterparts.

Important differences exist between this study and previous investigations of naloxone availability. Early studies of comparative survey methodologies demonstrated differences in response rates and content between telephonic and in-person surveys of household drug use,^25^ but few if any studies have described differences in healthcare professional responses to telephonic vs in-person queries of available services. It is likely that our results may have differed if we had contacted pharmacies by telephone, rather than presenting in person. Importantly, at least one point of contact with a community pharmacy led to the correction of a pharmacist’s erroneous belief that POSN was not available in her pharmacy. This community-based research, in which investigators sought to contribute to broader medical knowledge while at the same time effecting change to improve community health, is previously described,^26^ and represents a unique form of community advocacy undertaken at the level of the individual poison center.

The finding that up to 45% of pharmacy staff reporting no POSN availability were unaware of statutory support for collaborative prescribing protocols highlights two important findings from this study. First, an opportunity exists to better educate community pharmacists such that POSN is acknowledged as an option. When coupled with the community-based approach to survey data collection, this finding also provided an immediate opportunity to educate pharmacists at the point of contact regarding statutory support for POSN. Poison centers are well-known agents of change with respect to legislative lobbying;^27^ however, affecting change at the level of individual pharmacies through face-to-face interaction is unique even among poison centers. Although a national trend in increased naloxone prescriptions is evident, 2018 saw more than half a million prescriptions for naloxone written, compared to more than 38 million prescriptions for high-dose opioids.^28^ An opportunity exists to augment naloxone dispensation relative to opioid prescriptions; poison centers may hold multiple roles in this endeavor.

In addition to the isolated finding of a pharmacist prompted to revisit retail protocols to verify POSN availability despite her previous understanding to the contrary, still other reported reasons for failure to provide POSN were uncovered. These ranged from a perceived need to demonstrate overdose risk factors prior to providing POSN, to using personal discretion in deciding not to provide POSN despite an acknowledged capacity and institutional policy to provide it. These responses highlight opportunities for additional education within pharmacies to optimize naloxone distribution. Although assessing the impact of this survey on naloxone availability was not a formal study aim, future investigations might consider iterative assessments of naloxone availability following similar surveys.

## LIMITATIONS

A number of limitations apply to this study. Of particular note, we sampled a fixed, random sample of pharmacies within the metropolitan area. Although nearly one out of every five community pharmacies were successfully approached, a broader sample would have added strength to our findings. However, the in-person approach to surveying resulted in successful assessments of all pharmacies included in the study, and thus our sample likely represents as good or better an appraisal as a telephonic survey would have, accounting for likely non-responders. Nonetheless, our study did not account for medication stocking maintenance or other barriers to dispensation previously reported to affect individual pharmacies’ capacities to provide POSN.^29^

Similarly, our resources limited us from investigating naloxone availability within a broader geographic region including more rural communities. Our findings are thus more limited in their generalizability. Nonetheless, the finding that independent pharmacies are less likely to provide POSN, contextualized in previous data suggesting that rural areas in the region are heavily served by independent pharmacies, suggests that rural regions are less likely to have access to POSN via collaborative prescription protocols. An additional geographic limitation of this study was the specificity of our findings to a single state. Legislative efforts to promote POSN availability are variable across states, impacting state-to-state availability of POSN. Differences in availability of POSN secondary to legislative differences would likely be found by the current study protocol, given the “boots on the ground” approach to data collection; however, the evaluation of state-to-state differences in POSN availability was beyond the scope of the current study.

We chose to oversample independent pharmacies intentionally, at the expense of chain pharmacies. While this may have introduced a degree of bias to results, oversampling of independent pharmacies provided for a more balanced population and assessment of the impact of pharmacy type on POSN availability, an association that we predicted based on previously published research. Ultimately this decision likely led to an underestimate of POSN availability in our community, although our sensitivity analysis suggested a magnitude of underestimation to be approximately 5%.

Finally, we did not collect data on the role of the employee approached at individual pharmacies. It is plausible that answers would differ meaningfully between pharmacy technicians and pharmacists. We attempted to address this possible confounder by following up negative responses delivered by non-pharmacists with requests to speak directly with a pharmacist. Delivering the study question to the pharmacy professional who greeted researchers, best reflecting actual conditions at the point of sale, was determined to be a better reflection of reality than directing the study question solely to pharmacists.

## CONCLUSION

Point-of-sale naloxone is more widely available in this Midwestern metropolitan area than in recently described metropolitan areas in other regions of the United States. Although collaborative prescribing protocols are one of many naloxone distribution strategies contributing to harm reduction efforts, the survey method used in this study represents a unique “boots on the ground” for poison center professionals to champion change.

## Figures and Tables

**Table 1:** Characteristics of pharmacies.

Pharmacies	N (%)
Number sampled	150 (100)
Excluded *a priori* or at time of assessment	52 (34.7)
Included for analysis	98 (65.3)
Urban	75 (76.5)
Rural	23 (23.5)
Chain	72 (73.5)

**Table 2:** Comparison of point-of-sale naloxone availability by pharmacy characteristic.

Pharmacy characteristic	POSN N (%)	P-value
Chain	61/72 (84.7)	
Independent	10/26 (38.5)	<0.001
Urban	56/75 (74.7)	
Rural	15/23 (65.2)	0.375

*POSN*, point-of-sale naloxone.

**Table 3:** Point-of-sale naloxone availability across chain pharmacies with more than one site sampled.

Pharmacy:	A	B	C	D	E	F	G
n	18/19	15/16	7/9	7/8	7/7	2/3	2/2
%	94.7	93.8	77.8	87.5	100	66.7	100

**Table 4:** Awareness of state statutory support for point-of-sale naloxone among pharmacists reporting no access to point-of-sale naloxone.

Pharmacy characteristic	POSN N(%)	P-value
Chain	17/27 (63.0)	
Independent	11/16 (68.8)	0.687
Urban	12/19 (63.2)	
Rural	5/8 (62.5)	1.000

*POSN*, point-of-sale naloxone.
